# Age-Specific Risk Factors for Cancer in a Long-Term Korean Cohort of Patients with Ankylosing Spondylitis Treated with TNF Inhibitors

**DOI:** 10.3390/jcm14227959

**Published:** 2025-11-10

**Authors:** Yeo-Jin Lee, Minji Kim, Soo Min Ahn, Seokchan Hong, Ji Seon Oh, Chang-Keun Lee, Bin Yoo, Yong-Gil Kim

**Affiliations:** 1 Department of Rheumatology, Asan Medical Center, University of Ulsan College of Medicine, Seoul 05505, Republic of Korea; 2 Department of Biochemistry and Molecular Biology, Asan Medical Center, University of Ulsan College of Medicine, Seoul 05505, Republic of Korea; 3 Department of Information Medicine, Big Data Research Center, Asan Medical Center, Seoul 05505, Republic of Korea

**Keywords:** malignancy, tumor necrosis factor inhibitors, ankylosing spondylitis

## Abstract

**Background/Objectives**: Tumor necrosis factor (TNF) inhibitors are widely used in ankylosing spondylitis (AS), but data on cancer risk in this young population remain limited. We aimed to identify factors associated with cancer during TNF inhibitor therapy and evaluate age-specific risks in Asian patients with AS. **Methods**: We analyzed 810 AS patients who initiated TNF inhibitors between 2003 and 2023 at a Korean tertiary center. Cox regression assessed cancer predictors; when sparse data limited conventional models, Firth’s penalized likelihood was applied. Kaplan–Meier curves compared cumulative cancer incidence by age. **Results**: Over a mean follow-up of 7 years, cancer incidence was 323 per 100,000 person-years. Patients with cancer were older (48.6 vs. 39.0 years, *p* = 0.002) and more likely to have hypertension (33.3% vs. 14.6%, *p* = 0.041). In multivariable analysis, only age remained significant, with each year increasing hazard by 5.7%. Stratified analyses showed rising risks: HR 2.0 (≥30 vs. <30 years), 3.6 (≥40), 6.0 (≥50), and 8.8 (≥60). Risk profiles differed by age: in patients aged <40 years, female sex and elevated ESR were associated with cancer, while in patients aged ≥40 years, only age was associated. Thyroid cancer predominated in younger patients and lung cancer in older patients. **Conclusions**: In this long-term Korean cohort of patients with AS treated with TNF inhibitors, age was the primary independent risk factor, though determinants varied across age groups. These findings underscore the need for age-specific cancer risk assessment in AS.

## 1. Introduction

Ankylosing spondylitis (AS) is a chronic rheumatic disease that predominantly affects men in their 30s and 40s, often during their most productive working years [1,2]. Since the U.S. Food and Drug Administration (FDA) approved etanercept for AS in 2003 [3], tumor necrosis factor (TNF) inhibitors have been widely used in disease management. In Korea, etanercept (2003), infliximab (2006), adalimumab (2007), and golimumab (2013) became available and have been reimbursed for patients with radiographically confirmed sacroiliitis of grade ≥ 2. Despite the development of newer biologics, TNF inhibitors remain the most widely used and most commonly prescribed first-line biologic agents for AS [4].

When TNF inhibitors were first introduced, concerns arose regarding a potential increase in cancer risk [5]. This was based on the role of TNF-α in host defense, particularly in infection control and tumor surveillance via natural killer cells and CD8+ lymphocytes [6]. Consequently, numerous studies initially examined this association in rheumatoid arthritis (RA), where TNF inhibitors had been introduced earlier, but the findings were inconsistent [7,8,9].

Compared with RA, AS typically manifests at a younger age, which may lessen immediate concerns regarding cancer development. Nonetheless, several studies have reported an increased risk of cancer in patients with AS compared with the general population [10,11], with some suggesting the association may be more pronounced in Asian populations than in Western cohorts [12]. However, evidence directly assessing the relationship between TNF inhibitor therapy and cancer in AS remains limited. Early investigations were largely based on patient data collected from randomized controlled trials with relatively short treatment durations of 1–2 years [13,14]. More recently, a large study from Sweden and Denmark reported no increased cancer risk associated with TNF inhibitor use in patients with spondyloarthritis or psoriatic arthritis [15]. Notably, cancer incidence and type vary by geography and ethnicity. For instance, gastric and liver cancers are more common in East Asia, while prostate and colorectal cancers predominate in Western countries [16]. Thus, findings from Western registries may not be directly applicable to Asian populations. Given these regional differences and the relatively young age of Asian patients with AS, this study aimed to evaluate age-specific cancer risks associated with TNF inhibitor therapy and to identify factors contributing to malignancy development in Korean patients with AS.

## 2. Materials and Methods

### 2.1. Study Design and Population

**Figure 1:**
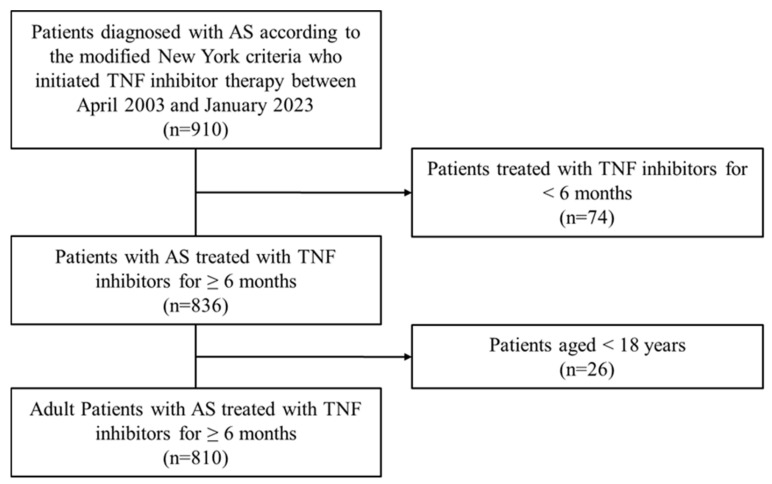
Flowchart illustrating patient selection.

This real-world retrospective study included patients aged ≥18 years who were diagnosed with AS and initiated TNF inhibitor therapy between April 2003 and January 2023 at a single tertiary referral center in Seoul, South Korea. Patients who used TNF inhibitors for <6 months were excluded. Follow-up continued until December 2023. For patients who switched from one TNF inhibitor to another, treatment was regarded as continuous TNF inhibitor exposure. Patients who discontinued therapy or were lost to follow-up were censored at the date of discontinuation or their last clinical visit, whichever occurred first. Regarding off-drug person-time, patients who discontinued TNF inhibitor therapy but restarted it within 3 months were considered to have maintained continuous exposure, consistent with criteria applied in previous cancer-related studies [17]. In contrast, if therapy was not resumed within 3 months, the off-drug period was excluded from the TNF inhibitor–exposed person-years. A flow diagram illustrating patient selection and exclusion is provided in Figure 1. The diagnosis of AS was confirmed according to the Modified New York criteria [18]. In accordance with Health Insurance Review and Assessment Service coverage criteria, TNF inhibitors were prescribed to patients with a Bath Ankylosing Spondylitis Disease Activity Index (BASDAI) score ≥ 4 despite prior nonsteroidal anti-inflammatory drug treatment. This study was conducted in accordance with the Declaration of Helsinki and its subsequent amendments. The Institutional Review Board of Asan Medical Center approved the study protocol (IRB No. 2015-0274), which was initially approved on 20 March 2015 and has been reviewed and renewed annually thereafter. Owing to the retrospective design, the requirement for informed consent was waived.

### 2.2. Data Collection

Baseline characteristics at the time of TNF inhibitor initiation were obtained from electronic medical records. These included age, sex, HLA-B27 status, comorbidities (hypertension and diabetes mellitus), smoking status, history of malignancy, BASDAI score, and laboratory markers (erythrocyte sedimentation rate [ESR] and C-reactive protein [CRP]). Treatment-related variables collected were the type of TNF inhibitor, duration of TNF inhibitor use, and time from AS diagnosis to TNF inhibitor initiation. Former and current smokers were classified as smokers. Malignancy events were identified through a comprehensive review of the institutional electronic medical records, including pathology reports, imaging findings, and clinical documentation. Cancer diagnoses were confirmed by board-certified physicians and recorded as the date of initial diagnosis. Concomitant medications were defined as agents that were co-administered with TNF inhibitors for ≥1 month.

### 2.3. Statistical Analysis

Categorical variables were summarized as counts and percentages (*n*, %), and continuous variables as means with standard deviations (SDs) or medians with interquartile ranges (IQRs). Group comparisons were performed using the Chi-squared test or Fisher’s exact test for categorical variables, the independent *t*-test for normally distributed continuous variables, and the Mann–Whitney U test for non-normally distributed continuous variables. Factors associated with cancer occurrence during TNF inhibitor therapy were evaluated using Cox proportional hazards regression. For variables with sparse data, Firth’s penalized likelihood method was applied. Variables significant in the univariate analyses were entered into multivariable models. Results were expressed as hazard ratios (HRs) with 95% confidence intervals (CIs). All analyses were conducted using SPSS version 21.0 (IBM, Armonk, NY, USA) and R version 4.3.3 (R Foundation for Statistical Computing, Vienna, Austria) via the RStudio interface (version 2023.6.1.524, Posit Software, Boston, MA, USA). Kaplan–Meier survival curves were generated in R. A *p*-value of <0.05 was considered statistically significant. Missing laboratory data were excluded from their respective analysis.

## 3. Results

### 3.1. Baseline Characteristics

During the study period, 910 patients with AS initiated TNF inhibitor therapy (adalimumab, etanercept, infliximab, or golimumab). Of these, 74 were excluded due to treatment duration <6 months, and 26 for being <18 years of age, leaving 810 patients for analysis. Adalimumab was the most frequently prescribed first-line agent (*n* = 447, 55.2%), followed by etanercept (25.1%), infliximab (12.1%), and golimumab (7.7%). As a second-line agent, adalimumab was also the most common (*n* = 70, 38.0%), followed by etanercept (28.8%), golimumab (19.0%), and infliximab (14.1%). The total follow-up duration was 5570 person-years, during which 18 incident cancers were identified (323 per 100,000 patient-years). As shown in Table 1, the mean age of patients at TNF inhibitor initiation was 39.2 ± 13.0 years; 75.9% were male, 90.1% were HLA-B27-positive, approximately half were ever smokers, and 2.8% had a history of cancer. The mean duration of TNF inhibitor therapy was 82.7 ± 60.9 months. Patients who developed cancer were older (48.6 vs. 39.0 years, *p* = 0.002) and more likely to have hypertension (33.3% vs. 14.6%, *p* = 0.041).

### 3.2. Characteristics and Outcomes of Malignancies

Among the 18 patients who developed cancer, the mean duration of TNF inhibitor exposure before cancer diagnosis was 103.8 ± 50.7 months (Table 2). The most frequent cancer types were lung cancer (*n* = 4), thyroid cancer (*n* = 3), and colorectal cancer (*n* = 2). Stratified by age, lung cancer was the most frequent malignancy in patients aged ≥40 years, whereas thyroid cancer predominated in those aged <40 years. One patient with thyroid cancer and another with early-stage colorectal cancer resumed TNF inhibitor therapy after surgical resection due to persistent pain and uncontrolled disease activity; both remained recurrence-free during ≥5 years of follow-up. One patient diagnosed with acute myeloid leukemia expired during the observation period.

### 3.3. Factors Associated with Cancer Occurrence

Cox regression analysis was performed to identify factors associated with cancer occurrence during TNF inhibitor therapy (Table 3). In univariable analysis, older age, hypertension, diabetes, and a history of cancer were significantly associated with malignancy. In multivariable analysis, only age remained statistically significant, with each one-year increase conferring a 5.7% higher hazard of cancer.

**Figure 2:**
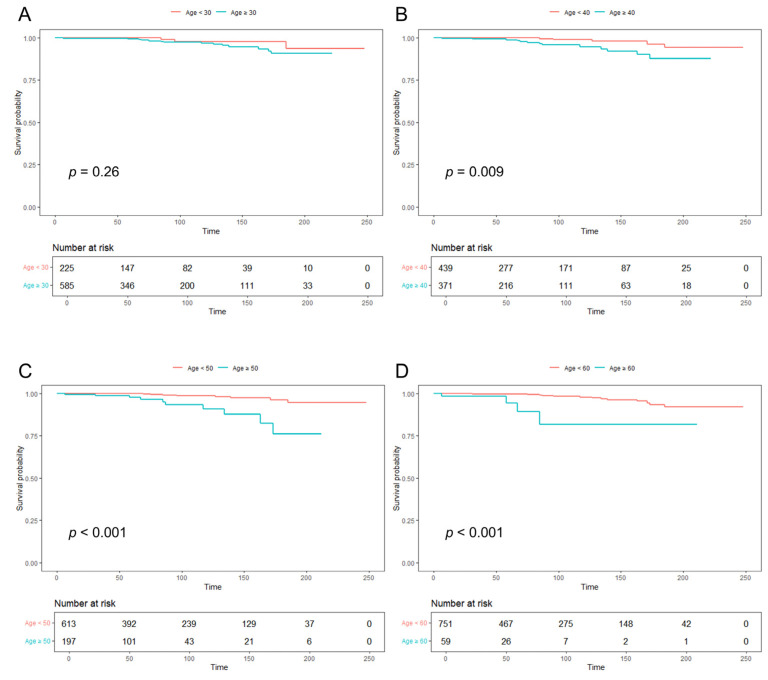
Kaplan–Meier curves of cancer occurrence in patients with AS treated with TNF inhibitors, stratified by age group: (**A**) <30 vs. ≥30 years, (**B**) <40 vs. ≥40 years, (**C**) <50 vs. ≥50 years, and (**D**) <60 vs. ≥60 years. AS, ankylosing spondylitis; TNF, tumor necrosis factor.

Kaplan–Meier analysis revealed no difference in cancer risk at the 30-year age cutoff, whereas risk was significantly higher in patients aged ≥40, ≥50, and ≥60 years compared with younger counterparts (*p* = 0.009, *p* < 0.001, and *p* < 0.001, respectively) (Figure 2). Corresponding hazard ratios were 2.013 (95% CI, 0.583–6.955) for ≥30 vs. <30 years, 3.624 (95% CI, 1.291–10.170) for ≥40 vs. <40 years, 5.978 (95% CI, 2.348–15.216) for ≥50 vs. <50 years, and 8.762 (95% CI, 2.812–27.299) for ≥60 vs. <60 years.

To further explore age-specific risk factors, patients were stratified at the 40-year cutoff, which closely approximated the median age of the cohort (38 years) and corresponded to a point where cancer risk began to increase significantly in the Kaplan–Meier analyses (Table 4). In those aged <40 years, female sex and elevated ESR were associated with increased cancer risk. In contrast, among patients aged ≥40 years, only age itself remained a significant risk factor.

## 4. Discussion

In this retrospective cohort of patients with AS treated with TNF inhibitors and followed for a mean duration of seven years, the overall cancer incidence was 323 per 100,000 person-years. Age was the only independent risk factor for cancer, with Cox regression analyses showing a stepwise increase in risk across age groups. When stratified at the 40-year cutoff, risk factors differed by subgroup: female sex and elevated ESR were associated with cancer in younger patients, whereas age alone was significant in older patients. The strengths of this study include the long follow-up period, its focus on an Asian AS cohort—where long-term data remain limited—and the exploration of age-specific differences in cancer risk.

Given that TNF-α blockade is central to AS management, understanding the dual role of TNF-α in tumor biology provides important context for interpreting these findings. TNF-α plays a complex, context-dependent role in tumor biology, functioning as both a tumor suppressor and promoter. Physiologically, TNF-α contributes to tumor surveillance by inducing apoptosis of malignant cells and enhancing cytotoxic T-cell and natural killer cell activity through TNFR1-mediated signaling pathways [19,20]. However, chronic TNF-α exposure within inflammatory environments can paradoxically promote carcinogenesis by sustaining NF-κB activation, enhancing angiogenesis, and inducing DNA damage, thereby facilitating malignant transformation and tumor progression [21]. This dual role of TNF-α underscores the need to balance its pro-inflammatory and anti-tumor functions when evaluating the long-term cancer risk associated with TNF inhibitor therapy in autoimmune diseases [22].

The relationship between TNF inhibitor therapy and cancer development in Asian patients with AS remains insufficiently studied. In Taiwan, nationwide registry studies comparing patients with AS to the general population reported that AS itself was associated with increased cancer risk; however, patients treated with TNF inhibitors were not analyzed separately [23,24]. Evidence from RA cohorts offers indirect insight: a recent Japanese registry study found that cancer incidence among TNF inhibitor users was comparable to that of the general population [25]. Although our analysis was limited to cancer incidence among patients receiving TNF inhibitors and therefore cannot determine whether TNF inhibitor exposure increases risk compared with TNF inhibitor-naïve patients, comparisons with existing Korean data provide context. In a nationwide cohort of AS patients with a mean age of 39.4 years and a mean follow-up of 3.3 years, cancer incidence was reported as 777 per 100,000 person-years (Supplementary Figure S1) [26]. Despite a comparable mean age, the incidence observed in our TNF inhibitor-treated cohort was lower, suggesting that TNF inhibitor therapy does not confer an excess cancer risk. Furthermore, our findings are consistent with registry-based studies from Western countries, which similarly reported no increased cancer risk with long-term TNF inhibitor use in patients with AS [15].

Among the potential risk factors evaluated, only age showed a significant independent association with cancer occurrence during TNF inhibitor therapy. This finding is expected, as advancing age is a well-established determinant of cancer risk in the general population, with incidence rising exponentially over time [27,28]. Our results extend this observation to patients with AS receiving long-term TNF inhibitor therapy, indicating that age—rather than treatment exposure or disease activity—is the predominant driver of malignancy risk. These findings suggest that cancer surveillance strategies in AS should focus on older patients, while providing reassurance that TNF inhibitor use itself does not confer additional risk.

In the subgroup of patients younger than 40 years, elevated ESR was associated with subsequent malignancy, a finding that likely reflects the combined influence of chronic inflammation and surveillance bias rather than a direct causal relationship. Chronic systemic inflammation has been mechanistically linked to tumor initiation and progression through cytokine-driven DNA damage, angiogenesis, and immune dysregulation [29]. Population-based studies have shown that patients with AS exhibit a modestly increased overall cancer risk compared with the general population, suggesting a role of sustained inflammatory burden in carcinogenesis [24]. Although BASDAI scores were not significantly associated with malignancy risk in our cohort, ESR may better capture systemic inflammatory activity over time, particularly in younger patients who experience longer cumulative exposure to inflammation. Further, younger patients with persistently elevated ESR may need to undergo additional evaluations or health-screening examinations at other medical facilities, which could facilitate earlier detection of subclinical malignancy. Accordingly, though these findings should be interpreted as hypothesis-generating, suggesting that unexplained, persistently elevated ESR—especially in younger female patients—may warrant a lower threshold for targeted assessment to exclude occult malignancy.

In our cohort, thyroid cancer was the most common malignancy among patients aged <40 years, whereas lung cancer predominated in those aged ≥40 years. A similar pattern was observed in the general Korean population, with thyroid cancer being the most frequent malignancy in individuals aged <40 years and lung cancer in those aged ≥40 years, consistent with our AS cohort receiving TNF inhibitors (Supplementary Table S1) [30]. By contrast, data from the SEER Explorer database showed that between 2018 and 2022, thyroid cancer was the second most frequent malignancy after testicular cancer among individuals aged <40 years (excluding breast cancer in females). In individuals aged ≥40 years, prostate cancer was the most common (after breast cancer in females), followed by lung cancer [31]. These findings underscore regional and ethnic differences in cancer incidence patterns.

This study has several limitations. Although we evaluated cancer incidence in patients receiving long-term TNF inhibitor therapy and identified characteristics associated with cancer occurrence, a direct comparison with sex- and age-matched TNF inhibitor-naïve patients or with the general population would be required to determine whether the incidence is truly higher or lower and whether TNF inhibitor use itself influences risk. In addition, this was a single-center study; therefore, future large-scale investigations using multicenter registry cohorts are needed to validate our findings.

In this long-term Korean cohort of patients with AS treated with TNF inhibitors, age was the only consistent independent risk factor, and stratified analyses demonstrated that the factors associated with cancer occurrence differed between younger and older patients. These findings highlight the importance of age-specific risk assessment when evaluating malignancy risk in patients receiving TNF inhibitors and underscore the need for larger studies to confirm these observations.

## Figures and Tables

**Table 1:** Baseline characteristics of patients with AS treated with TNF inhibitors, stratified by the occurrence of cancer during treatment.

	Total (*n* = 810)	Cancer Occurrence		*p*-Value
		No (*n* = 792)	Yes (*n* = 18)	
Age at TNF inhibitor initiation, years	39.2 ± 13.0	39.0 ± 12.9	48.6 ± 13.7	0.002
Male, *n* (%)	615 (75.9)	601 (75.9)	14 (77.8)	>0.999
HLA-B27, *n* (%)	715 (90.1)	698 (89.9)	17 (94.4)	>0.999
HTN, *n* (%)	122 (15.1)	116 (14.6)	6 (33.3)	0.041
DM, *n* (%)	37 (4.6)	35 (4.4)	2 (11.1)	0.197
Smoker, *n* (%)	433 (54.0)	422 (53.8)	11 (61.1)	0.540
History of cancer, *n* (%)	23 (2.8)	21 (2.7)	2 (11.1)	0.090
Duration of TNF inhibitor, months	82.7 ± 60.9	82.0 ± 61.0	110.1 ± 52.4	0.053
Duration from diagnosis of AS to TNF inhibitor initiation, months	9.0 (3.0–47.3)	9.0 (3.0–46.0)	29.0 (9.5–63.3)	0.113
BASDAI	6.8 ± 1.3	6.7 ± 1.3	7.2 ± 1.6	0.159
ESR, mm/h	40.1 ± 29.7	39.9 ± 29.6	50.4 ± 35.4	0.135
CRP, mg/dL	1.1 (0.4–2.3)	1.1 (0.4–2.3)	0.8 (0.5–2.1)	0.939
Concomitant treatment				
NSAIDs, *n* (%)	721 (89.0)	704 (88.9)	17 (94.4)	>0.999
csDMARDs, *n* (%)	222 (27.4)	214 (27.0)	8 (44.4)	0.255
Corticosteroid, *n* (%)	154 (19.0)	149 (18.8)	5 (27.8)	0.502
Cumulative corticosteroid dose, mg	0 (0–0)	0 (0–0)	0 (0–492.5)	0.312

Data are presented as mean ± standard deviation, median (interquartile range), or *n* (%). AS, ankylosing spondylitis; TNF, tumor necrosis factor; HLA, human leukocyte antigen; HTN, hypertension; DM, diabetes mellitus; BASDAI, Bath Ankylosing Spondylitis Disease Activity Index; ESR, erythrocyte sedimentation rate; CRP, C-reactive protein; NSAID, nonsteroidal anti-inflammatory drugs; csDMARDs, conventional synthetic disease-modifying anti-rheumatic drugs. Cumulative corticosteroid dose was expressed as prednisolone-equivalent (mg).

**Table 2:** Type of malignancy in patients with AS treated with TNF inhibitors.

	Age/Sex	Type of TNF Inhibitor and Exposure Period				Type of Malignancy
		1st Line	Months	2nd Line	Months	
1	32/M	Etanercept	171			Renal cell cancer
2	52/M	Etanercept	134			Acute myeloid leukemia †
3	50/M	Etanercept	173			Multiple myeloma
4	28/M	Etanercept	185			Thyroid cancer
5	59/M	Etanercept	117			Hepatocellular carcinoma
6	27/F	Etanercept	85			Gastric cancer
7	69/M	Etanercept	85			Pancreatic cancer
8	49/M	Adalimumab	75			Colorectal cancer
9	25/M	Adalimumab	96			Thyroid cancer *
10	44/F	Adalimumab	69			Endometrial cancer
11	62/M	Adalimumab	67			Lung cancer
12	65/M	Adalimumab	58			Lung cancer
13	58/M	Adalimumab	31			Lung cancer
14	51/M	Infliximab	87			Colon cancer *
15	60/F	Golimumab	6			Breast cancer
16	58/M	Etanercept	28	Infliximab	135	Lung cancer
17	37/F	Adalimumab	4	Etanercept	123	Thyroid cancer
18	48/M	Adalimumab	126	Etanercept	13	Lymphoma

* Restarted TNF inhibitor after treatment of malignancy. † Expired: 2 years 6 months after diagnosis.

**Table 3:** Factors associated with cancer occurrence in patients with AS treated with TNF inhibitors.

	Unadjusted HR (95% CI)	*p*-Value	Adjusted HR (95% CI)	*p*-Value
Age, years	1.074 (1.036–1.114)	<0.001	1.057 (1.014–1.102)	0.009
Male	0.685 (0.223–2.100)	0.508		
HLA-B27	1.372 (0.182–10.336)	0.759		
HTN	3.827 (1.428–10.254)	0.008	1.851 (0.593–5.779)	0.289
DM	4.800 (1.092–21.103)	0.038	2.276 (0.484–10.702)	0.298
BASDAI	0.966 (0.661–1.411)	0.858		
Smoker	1.075 (0.416–2.778)	0.882		
History of cancer	6.469 (1.484–28.205)	0.013	3.024 (0.653–14.003)	0.157
Duration from diagnosis of AS to TNF inhibitor initiation, months	1.002 (0.996–1.009)	0.494		
ESR, mm/h	1.007 (0.993–1.022)	0.332		
CRP, mg/dL	0.985 (0.789–1.229)	0.892		
Concomitant treatment				
NSAIDs	1.095 (0.144–8.354)	0.930		
csDMARDs	1.128 (0.440–2.889)	0.802		
Cumulative corticosteroid dose, mg	1.000 (1.000–1.000)	0.869		

AS, ankylosing spondylitis; TNF, tumor necrosis factor; HLA, human leukocyte antigen; HTN, hypertension; DM, diabetes mellitus; BASDAI, Bath Ankylosing Spondylitis Disease Activity Index; ESR, erythrocyte sedimentation rate; CRP, C-reactive protein; NSAID, nonsteroidal anti-inflammatory drugs; csDMARDs, conventional synthetic disease-modifying anti-rheumatic drugs; HR, hazard ratio; CI, confidence interval. Cumulative corticosteroid dose was expressed as prednisolone-equivalent (mg).

**Table 4:** Factors associated with cancer occurrence in patients with AS treated with TNF inhibitors, stratified by age group (<40 vs. ≥40 years).

	Unadjusted HR (95% CI)	*p*-Value	Adjusted HR (95% CI)	*p*-Value
Age <40 Years (*n* = 439)				
Age, years	1.010 (0.875–1.166)	0.893		
Male	0.104 (0.016–0.681)	0.018	0.098 (0.013–0.747)	0.025
HTN	1.479 (0.011–13.582)	0.803		
BASDAI	2.785 (0.973–7.973)	0.056		
Smoker	1.008 (0.168–6.032)	0.993		
History of cancer	38.089 (0.276–468.707)	0.108		
Duration from diagnosis of AS to TNF inhibitor initiation, months	1.006 (0.989–1.023)	0.514		
ESR, mm/h	1.034 (1.004–1.064)	0.026	1.035 (1.004–1.067)	0.026
CRP, mg/dL	1.286 (0.965–1.713)	0.086		
Concomitant treatment				
NSAIDs	0.697 (0.073–92.897)	0.818		
csDMARDs	2.080 (0.346–12.493)	0.423		
Cumulative corticosteroid dose, mg	1.000 (1.000–1.001)	0.265		
Age ≥40 years (*n* = 371)				
Age, years	1.092 (1.034–1.153)	0.002	1.074 (1.012–1.141)	0.019
Male	2.117 (0.467–9.591)	0.331		
HTN	3.395 (1.120–10.287)	0.031	2.049 (0.583–7.205)	0.263
DM	2.629 (0.579–11.927)	0.210		
BASDAI	0.767 (0.496–1.188)	0.236		
Smoker	1.106 (0.360–3.394)	0.860		
History of cancer	4.195 (0.928–18.965)	0.063		
Duration from diagnosis of AS to TNF inhibitor initiation, months	1.001 (0.994–1.008)	0.871		
ESR, mm/h	0.995 (0.977–1.013)	0.594		
CRP, mg/dL	0.816 (0.556–1.197)	0.298		
Concomitant treatment				
NSAIDs	0.521 (0.065–4.192)	0.540		
csDMARDs	0.824 (0.262–2.592)	0.741		
Cumulative corticosteroid dose, mg	1.000 (0.999–1.000)	0.618		

AS, ankylosing spondylitis; TNF, tumor necrosis factor; HTN, hypertension; DM, diabetes mellitus; BASDAI, Bath Ankylosing Spondylitis Disease Activity Index; ESR, erythrocyte sedimentation rate; CRP, C-reactive protein; NSAID, nonsteroidal anti-inflammatory drugs; csDMARDs, conventional synthetic disease-modifying anti-rheumatic drugs; HR, hazard ratio; CI, confidence interval. Cumulative corticosteroid dose was expressed as prednisolone-equivalent (mg).

## Data Availability

The datasets generated and analyzed during the current study are available from the corresponding author, Yong-Gil Kim, on reasonable request. Due to Institutional Review Board restrictions, the data are not publicly available, as they contain information that could compromise participant privacy.

## Supplementary Materials

The following supporting information can be downloaded at: https://www.mdpi.com/article/10.3390/jcm14227959/s1, Figure S1: Comparison of overall cancer incidence among patients with AS treated with TNF inhibitors, the Korean male AS registry cohort, and the general Korean population. Table S1: Number of cases and incidence rates of cancer according to age and location in 2022 from the Korea National Cancer Incidence Database.
