# Supplementary material for: Age-Specific Risk Factors for Cancer in a Long-Term Korean Cohort of Patients with Ankylosing Spondylitis Treated with TNF Inhibitors
Source: J Clin Med. 2025 Nov 10;14(22):7959. doi: 10.3390/jcm14227959 (PMC12653172; doi:10.3390/jcm14227959)
Supplement: Supplementary file 1 [file jcm-14-07959-s001.zip › jcm-3934861-supplementary.pdf]

Supplementary Table S1. Number of cases and incidence rates of cancer according to age and location in 2022 from the Korea National Cancer Incidence Database

	Age 0–39 years		Age ≥40 years	
	Cases, *n*	Crude incidence rate, per 100,000 persons	Cases, *n*	Crude incidence rate, per 100,000 persons
All cancers (C00–C96)	20,987	81.91	261,060	1398.58
Lip, oral cavity, and pharynx (C00–C14)	317	1.25	4,367	21.68
Esophagus (C15)	10	0.04	3,034	16.30
Stomach (C16)	488	1.81	28,999	158.69
Colon and rectum (C18–C20)	1,776	6.66	31,382	174.49
Liver (C22)	223	0.93	14,690	83.15
Gallbladder and other biliary tract (C23–C24)	30	0.11	7,818	55.18
Pancreas (C25)	219	0.88	9,561	59.63
Larynx (C32)	5	0.01	1,224	6.68
Lung (C33–C34)	302	1.14	32,011	198.90
Breast (C50)	2,129	7.94	27,399	102.91
Cervix uteri (C53)	544	2.03	2,630	11.50
Corpus uteri (C54)	405	1.53	3,553	13.28
Ovary (C56)	374	1.50	2,889	12.70
Prostate (C61)	4	0.00	20,750	129.19
Testis (C62)	239	0.94	116	0.45
Kidney (C64)	496	1.91	6,467	29.40
Bladder (C67)	44	0.16	5,217	34.79
Brain and central nervous system (C70–C72)	416	1.98	1,747	9.10
Thyroid (C73)	9,939	37.60	23,975	82.26
Hodgkin lymphoma (C81)	157	0.68	221	1.06
Non-Hodgkin lymphoma (C82–C86, C96)	585	2.44	5,484	29.98
Multiple myeloma (C90)	12	0.04	1,949	11.33
Leukemia (C91–C95)	798	3.85	3,071	16.39

Supplementary Figure S1. Comparison of overall cancer incidence among patients with AS treated with TNF inhibitors, the Korean male AS registry cohort, and the general Korean population.

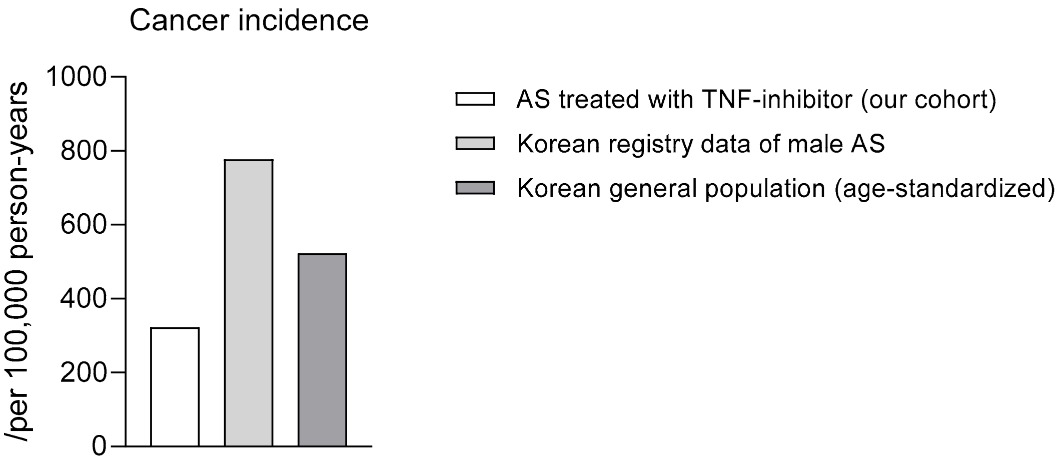

AS, ankylosing spondylitis; TNF, tumor necrosis factor. Data are presented for contextual comparison; no direct statistical testing was performed because the national registry data for the general population represent annual incidence, whereas the current study assessed longitudinal incidence over a mean follow-up period of seven years. In addition, the follow-up period of the nationwide AS cohort was shorter (mean 3.3 years), which may contribute to observed differences in incidence.
